# Endoscopic Treatment of Scaphocephaly: A Case Study

**DOI:** 10.7759/cureus.72246

**Published:** 2024-10-23

**Authors:** Virginie Lafontaine, Florian Le Lann, Laurane Benoit, Jean Philippe Giot, Béatrice Morand

**Affiliations:** 1 Plastic and Maxillofacial Surgery, Centre Hospitalier Universitaire (CHU) Grenoble Alpes, Grenoble, FRA; 2 Neurosurgery, Centre Hospitalier Universitaire (CHU) Grenoble Alpes, Grenoble, FRA; 3 Plastic Surgery, Centre Hospitalier Universitaire (CHU) Grenoble Alpes, Grenoble, FRA

**Keywords:** craniectomy, craniosynostosis surgery, endoscopic surgery, sagittal suture, scaphocephaly

## Abstract

Craniosynostosis is a congenital craniofacial anomaly caused by the premature closure of one or more sutures. Scaphocephaly is the most common form. Endoscopic surgery is increasingly being used for the treatment of a single synostosis. We present our first case of endoscopy-assisted surgery for scaphocephaly in a three-month-old baby. Follow-up at one year was satisfactory, showing a good aesthetic result. The child did not require blood transfusion. Endoscopic surgery presents various advantages in treating scaphocephaly. It is necessary to educate our primary care physicians and improve their awareness about the importance of endoscopic surgeries in scaphocephaly.

## Introduction

Craniosynostosis is a congenital craniofacial anomaly caused by the premature closure of one or more sutures. Craniosynostosis is a common condition that affects the neurocognitive and craniofacial skeletal development of children worldwide. The birth prevalence of non-syndromic craniosynostosis is 5.2 per 10,000 live births [1]. Scaphocephaly is the most common form and occurs in 40 to 60% of all craniosynostosis [2].

According to Virchow’s law, compensatory growth occurs in the direction parallel to the affected suture [3]. On physical examination, an elongated and narrowed head shape is found, characterized by frontal and occipital bossing and narrow biparietal distance [4].

The treatment is surgical. Cranial vault remodelling through large skin incisions has been used for decades [5]. The endoscopic techniques were introduced in the early 1990s by Jimenez and Barone [6]. Minimally invasive surgery is increasingly being used to treat single synostosis, but surgical techniques and patient management vary widely between centres [7].

Our department has a long tradition and extensive experience in the surgical treatment of craniosynostosis, particularly in open cranial vault remodelling. We present our first case of endoscopy-assisted surgery for scaphocephaly with a one-year follow-up.

## Case presentation

We present a case of a one-month-old boy referred to the multidisciplinary neurosurgical and maxillofacial consultation for suspected craniostenosis. The patient has no family history of craniostenosis. The baby was born at term, eutrophic with a cranial circumference of 35 cm. Clinically he presented with dolichocephaly with a sagittal crest and biparietal narrowing. Psychomotor development was age-appropriate with good weight gain.

**Figure 1:**
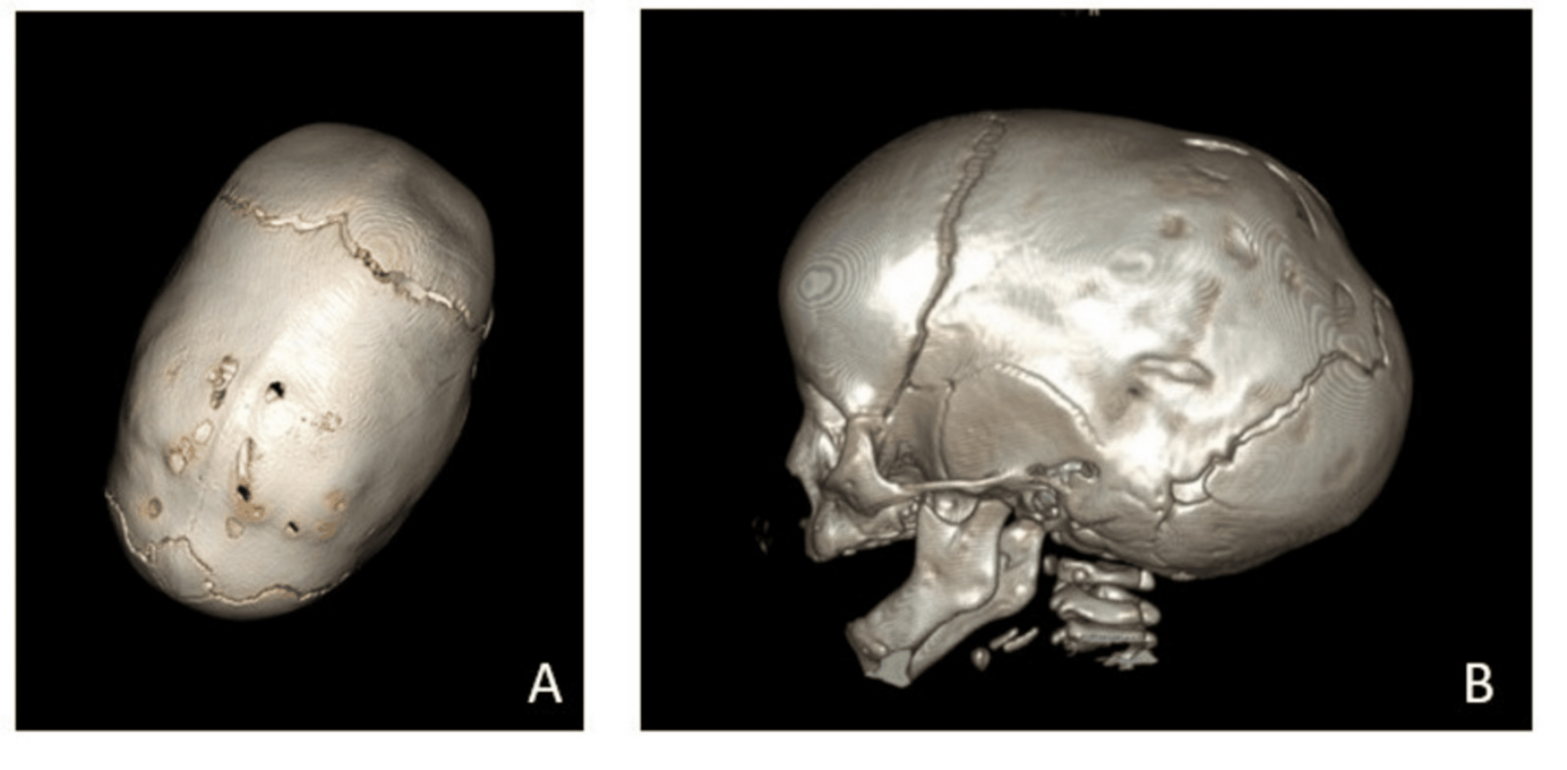
Pre-operative CT scan with reconstruction A: Vertex view; B: Lateral view

A CT scan confirmed complete closure of the sagittal suture (Figure 1). The fundus showed no intracranial hypertension.

**Figure 2:**
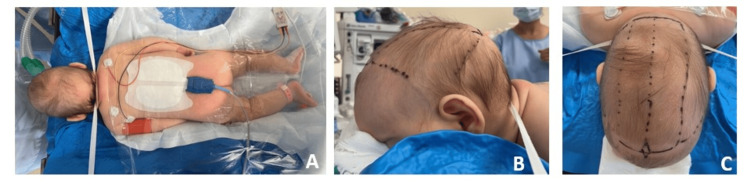
Surgical installation A: Total view in the sphinx position; B: Side view of head in the sphinx position; C: Landmarks and drawing incisions

Endoscopic surgery was performed at three months of age after using a pre-operative erythropoietin (EPO) home delivery protocol. Landmarks were drawn: lambda and bregma (fontanelle), coronal and lambdoid sutures, two transverse skin incisions of 4.5 cm opposite fontanelle (Figure 2). Before incision, the skin was infiltrated with xylocaine, adrenalin 1%. We performed subperiosteal detachment followed by burr holes with a high-speed drill and Kerrison in order to do craniectomy opposite incision.

**Figure 3:**
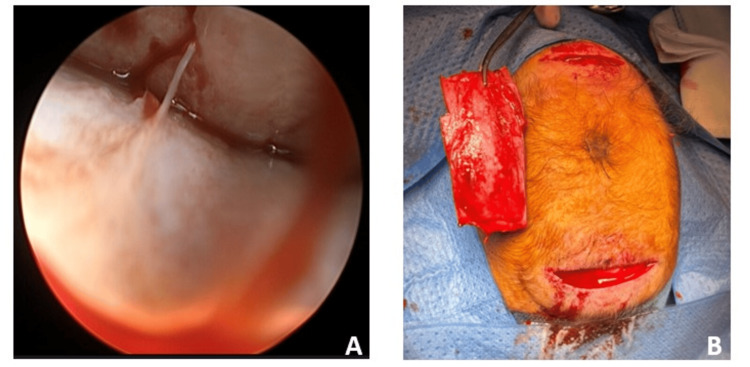
Intra-operative views A: Endoscopic view into the epidural space; B: Sagittal suturectomy

An endoscope was introduced into the epidural space. Sagittal suturectomy of 5 cm was done with scissors (Figure 3) like corticotomy in front of the lambdoid sutures and behind the coronals. Tisseel was introduced for hemostasis. In the end, the incisions were sutured without drainage.

The patient was admitted to the intensive care unit for 48 hours after surgery. Antibiotic prophylaxis with amoxicillin-clavulanic acid and methylprednisolone was administered for 48 hours. Blood transfusion was not required. The patient was then returned to the conventional surgical ward and discharged on day 5 post-op after follow-up with X-rays. No helmet therapy after surgery was required.

**Figure 4:**
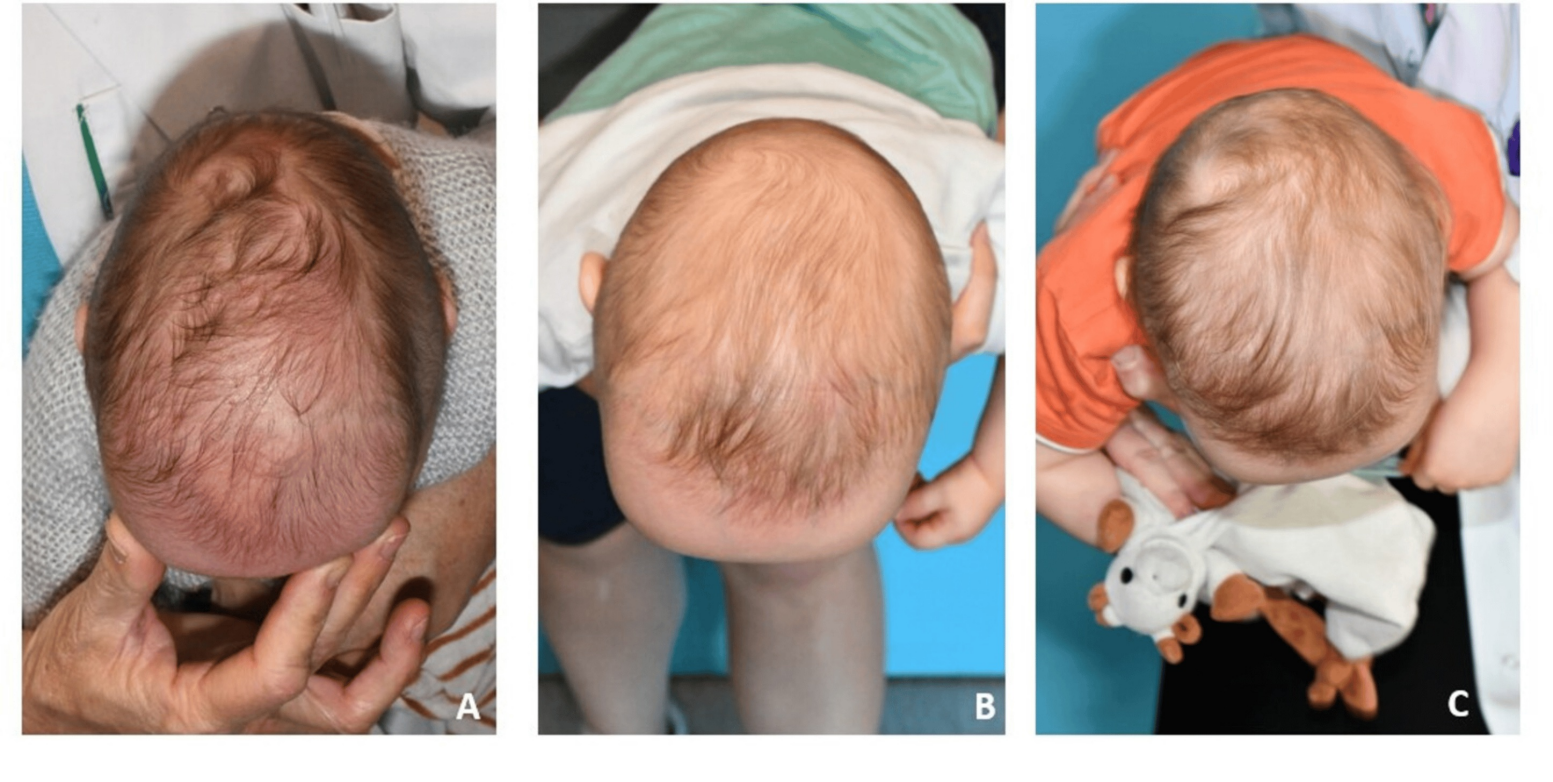
Top view of the vault A: Pre-operative; B: Three months after surgery; C: One year after surgery

Three-month post-operative follow-up showed good morphological correction with bi-parietal widening. One year after surgery, the biparietal width was good without bone defect (Figure 4). Psychological development was good with the acquisition of walking at 14 months.

## Discussion

Pre-operative examination

At our centre, patients are seen in a multidisciplinary consultation involving a neurosurgeon and a maxillofacial surgeon. Clinical examination often suggests a diagnosis of single-suture craniosynostosis. The pre-operative work-up always includes a CT scan combined with a funduscopic examination. If these examinations are carried out in the first few months of life, they can be done without general anaesthesia, but from the age of 3-4 months, they require sedation and monitoring for a few hours after the examination.

According to Swheitzer et al., a pre-operative CT scan can be avoided, as clinical examination and ultrasound in the first few months of life are sufficient to confirm the diagnosis [8]. A CT scan should be reserved for more complex forms where there is doubt about the suture(s) involved.

Funduscopy is not performed systematically by all teams. It is used to assess intracranial pressure by looking for papilledema, but also to look for possible deviations in line of sight [7].

Optimal age for the endoscopic-assisted craniosynostosis surgery

Endoscopic-assisted craniosynostosis surgery is generally reserved for small infants (<3 months) because of the thinness of their skull and the subsequent rapid brain growth, which allows for correction and normalisation of the craniofacial skeleton [9]. The ideal time for open surgery is 6-12 months [10,11]. This optimises re-ossification and bone remodelling and reduces the need for bone grafting.

In our centre, patients are often referred to us at 4-5 months of age. Thus, we are unable to schedule endoscopic surgery as soon as possible. To improve the way we treat these children, we are training our primary care physicians to inform the parents about the diagnosis early.

Choice of the surgical technique

The aim of surgery for sagittal suture craniosynostosis is to allow the brain to grow and remodel the skull vault by removing the blockage caused by the suture synostosis, widening the transverse direction and shortening the anteroposterior direction.

The first surgical treatment for sagittal craniosynostosis was performed by Lannelongue in 1892, with linear craniectomy [4,12]. At the time, craniosynostosis surgery was associated with a high risk of morbidity and mortality. It was abandoned for three decades. Over time, open surgery offers numerous osteotomy techniques ( “pi”, “H ”), sometimes reshaping the entire cranial vault [4,12]. The ideal treatment has not yet been found.

Endoscopic techniques have emerged in recent decades. Four categories of osteotomies were identified: strip craniectomy alone, strip craniectomy with wedges, strip craniectomy with parietal barrel staves and midline osteotomy with spring. The width of the osteotomy varies significantly between 1 and 7 cm [13].

Studies have shown comparable results between endoscopic surgery and open surgery in terms of head growth and aesthetic outcomes [14]. The size of the incisions is the only notable difference. In our case, the choice of technique is linked to the experience and the training that the surgeon received.

Assessment of the surgical technique

Meta-analyses demonstrate that open surgery takes longer than endoscopic surgery in craniosynostosis [15]. Thompson et al. reported medians of 1.16 hours for endoscopic procedures and 2.16 for open procedures [9]. Most studies do not distinguish anaesthesia preparation and surgery, the total time under general anaesthesia is evaluated [10]. Thompson et al. showed the preparation stage is longer in open surgery (130 min vs 70 min) [9].

A literature review showed that both intra-operative blood loss and intra-operative and post-operative blood transfusion volumes are lower in endoscopic surgery [10]. Endoscopic procedures showed a transfusion rate ranging from 0% to 26% of patients and open procedures ranged from 16% to 100% [15].

In our case, the patient had benefited from the pre-operative EPO protocol but did not require intra- or post-operative transfusion. This is in contrast to patients operated by the open technique at our centre who did receive post-operative blood transfusions. In fact, the learning curve has been shown to decrease surgical time and thus surgical bleeding [7].

Most studies show that the length of hospital stay is shorter with endoscopic surgery [10,15]. Thompson et al. conducted a multicenter retrospective study and reported a median of two days for endoscopic surgery versus four days for open surgery [9].

For the patient, as this was our first case, the duration of continuous monitoring and the total length of hospital stay were the same as those for open-surgery patients. In the future, our experience will enable us to reduce post-operative hospital stays and maybe avoid post-operative intensive care unit monitoring.

Hematoma, infection and scarring are the main complications of craniosynostosis surgery. Yan et al. in a meta-analysis study reported six studies with equal or lower rates of complications for endoscopically treated patients [15].

A meta-analysis of aesthetic outcomes showed similar results between open and endoscopic surgery for scaphocephaly [10]. Yan et al. reported three studies with a lower reoperation rate for endoscopic technique [15].

Studies have shown that the total cost of endoscopic surgery for sagittal synostosis is three times lower than that for open surgery. This is due to shorter hospital stays and lower transfusion rates for example.

## Conclusions

As a conclusion, we presented our first case of endoscopic scaphocephaly treatment. The aesthetic result at one year is satisfactory. The child did not require blood transfusion. Endoscopic surgery presents various advantages in treating scaphocephaly. In our centre, the aim is to reduce post-operative ICU monitoring with early hospital discharge. However, it is necessary to educate our primary care physicians and improve their awareness about the importance of endoscopic surgeries in scaphocephaly.
